# Novel variants in *AP4B1* cause spastic tetraplegia, moderate psychomotor development delay and febrile seizures in a Chinese patient: a case report

**DOI:** 10.1186/s12881-020-0988-3

**Published:** 2020-03-14

**Authors:** Wen-Cong Ruan, Jia Wang, Yong-Lin Yu, Yue-Ping Che, Li Ding, Chen-Xi Li, Xiao-Dong Wang, Hai-Feng Li

**Affiliations:** 1 Department of Rehabilitation, The Children’s Hospital, Zhejiang University School of Medicine, Zhejiang, 310052 China; 2 Cipher Gene, LLC, Beijing, 100080 China

**Keywords:** Spastic tetraplegia, Sequencing, Mutation, Rehabilitation

## Abstract

**Introduction:**

The *AP4B1* gene encodes a subunit of adaptor protein complex-4 (AP4), a component of intracellular transportation of proteins which plays important roles in neurons. Bi-allelic mutations in *AP4B1* cause autosomal recessive spastic paraplegia-47(SPG47).

**Case presentation:**

Here we present a Chinese patient with spastic tetraplegia, moderate psychomotor development delay and febrile seizures plus. Brain MRIs showed dilated supratentorial ventricle, thin posterior and splenium part of corpus callosum. The patient had little progress through medical treatments and rehabilitating regimens. Whole exome sequencing identified novel compound heterozygous truncating variants c.1207C > T (p.Gln403*) and c.52_53delAC (p.Cys18Glnfs*7) in *AP4B1* gene. Causal mutations in *AP4B1* have been reported in 29 individuals from 22 families so far, most of which are homozygous mutations.

**Conclusions:**

Our study enriched the genetic and phenotypic spectrum of SPG47. Early discovery, diagnosis and proper treatment on the conditions generally increase chances of improvement on the quality of life for patients.

## Introduction

Hereditary spastic paraplegias (HSPs) are a group of clinically and genetically heterogeneous neurological disorders with the features of progressive weakness and spasticity of lower limbs. Autosomal recessive HSPs are usually accompanied by other abnormalities such as seizures, intellectual disability, peripheral neuropathy, and/or extrapyramidal involvement [1]. The *AP4B1* gene encodes a subunit of adaptor protein complex-4(AP4), which is a component of intracellular transportation proteins [2, 3]. Four subunits of AP4 (*AP4M1*, *AP4E1*, *AP4S1*, and *AP4B1*) have been associated with similar autosomal recessive-HSP characterized by progressive spastic paraplegia and severe mental retardation with poor or absent speech development. These HSPs are collectively called “AP-4 deficiency syndrome” [4]. Bi-allelic mutations in *AP4B1* cause autosomal recessive spastic paraplegia-47 (SPG47, MIM: 614066). Disease-causing mutations in *AP4B1* have been reported in 29 individuals from 22 families. Most of these mutations are homozygous (23/29) [5–8].

Here we report novel compound heterozygous truncating variants in *AP4B1* in a nine years-old Chinese boy with clinical features including spastic tetraplegia, moderate psychomotor development delay, and febrile seizures plus (FS). The conditions were improved through several years of rehabilitation.

## Case presentation

### Clinical presentation

Patient is a 9-year-old boy born to non-consanguineous healthy parents. He was born at 40 weeks of gestation by natural delivery. The birth weight was 3.4 kg. Apgar scores were 10–10 − 10. There was no special medical history during pregnancy, and no perinatal complications were noticed. He was able to hold up his head firmly at 3 months, roll over at 6 months, sit uprightly on his own at 10 months, stand unaidedly at 20 months, and walk well at 2-year-old. He began to speak a few words at 2 years old, such as “baba”, “mama”.

He was admitted to the hospital at the age of 9 months for sitting unstably without assistance. The psychomotor developmental delay was noticed. Long-term local rehabilitation started immediately. During the time, he had a seizure triggered by fever. The conditions, including upward rolling of the eyes, lips cyanosis, tonic stiffening of the upper limbs and lacking of consciousness, lasted for almost 10 mins. He was diagnosed as febrile seizures plus (FS+) by his physician in local hospital. The patient was treated with oral administration of topiramate tablets for 3 months (dosage unknown). However, he suffered recurrent febrile seizures (2~3 times /year, lasting 2 to 10 min each time) with the same manifestations. At the age of 6, he was given 0.1 ml/kg (Bid) of oral solution of levetiracetam (100 mg/ml). One year later, seizures occurred again, but had been controlled with adjusted dosage to12.5 mg/kg (Bid) of levetiracetam tablets.

The patient presented unstable walk with lisping at seven-year-old. He was referred to a pediatric neurologist. His physical examination results were described as following: he could communicate and express his needs in simple words with a lisp; he could stand and walk without support, jump on both feet, and stand on one foot for a while. However, he could neither jump on one foot nor run. He appeared lumbar lordosis, pelvic tilt, hip flexion, foot valgus, insufficient camptodactyly of ankle when walking alone, bilateral babinski sign (+), and ankle clonus (+).

**Fig. 1:**
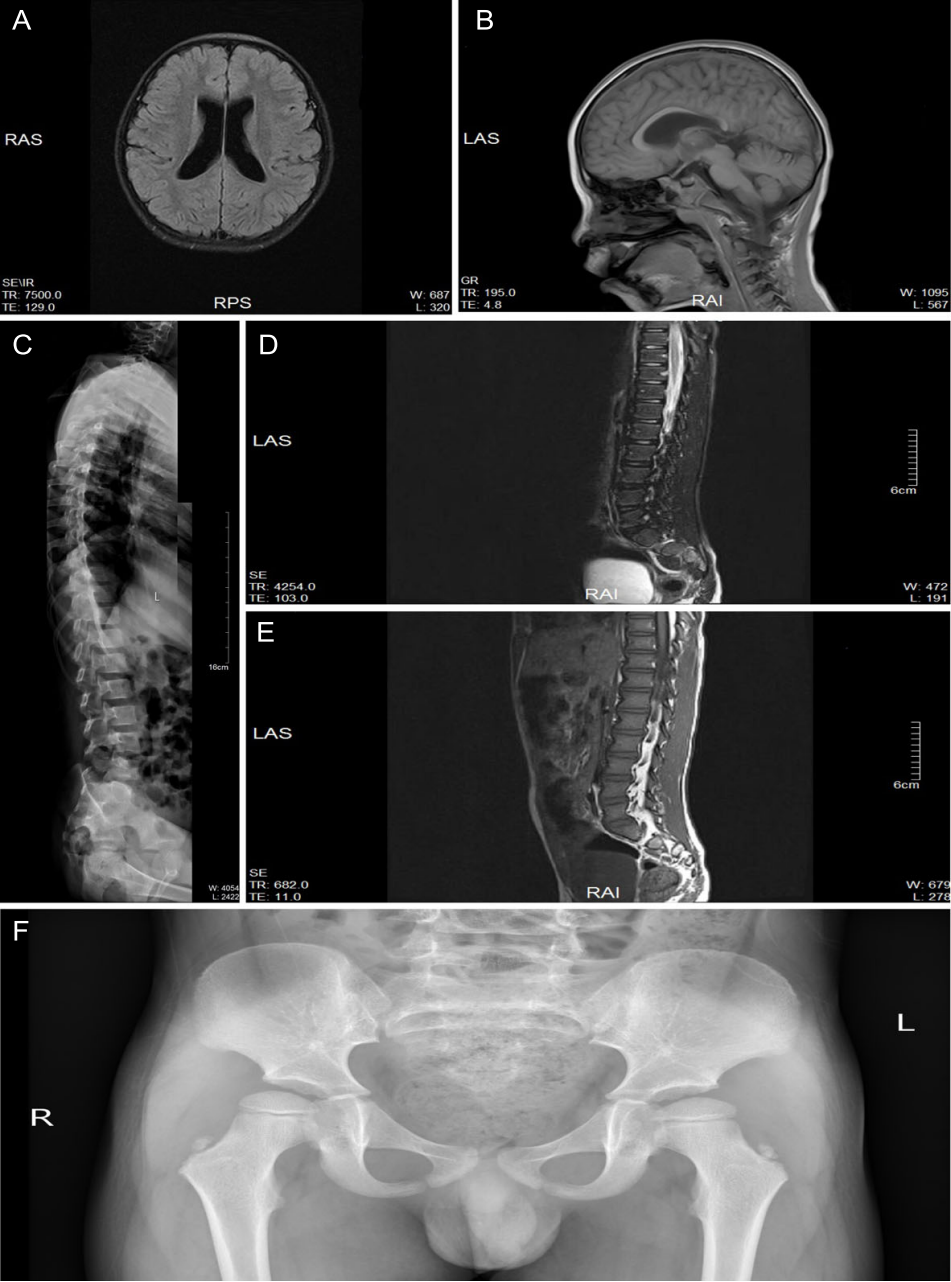
MRI scan results of patient presented unstable walk with lisping at seven-year-old and was referred to a pediatric neurologist at the 7-year-old (201702). **a** Brain MRIs showed dilated supratentorial ventricle. **b** Thin posterior part and splenium of corpus callosum. **c** Total spine MR imaging suggested 1, L4, 5 recessive spina bifida. **d** Cervical and thoracic spinal cord scan was normal. **e** Lumbosacral segment of spinal cord scan was normal. **f** Pelvic radiograph was normal

Brain MRIs showed dilated supratentorial ventricle (Fig. 1a), thin posterior and splenium part of corpus callosum (Fig. 1b). Total spine MR imaging suggested 1, L4, 5 recessive spina bifida (Fig. 1c). Cervical, thoracic, and lumbosacral segment of spinal cord scan, pelvic radiograph, and electromyography were all normal (Fig. 1d-f). Recessive spina bifida was the result of full (or lateral) splicing of the spine, and tethered spinal cord syndrome was excluded. No apparent changes were found from laboratory tests of blood routine, blood biochemistry, blood genetic metabolic mass spectrometry and thyroid functional indices.

### Molecular studies

We performed Whole Exome Sequencing on the patient in order to identify the disease-causing variants. The exomes were captured from peripheral blood DNA using Agilent SureSelectV6 kit and sequenced by Illumina HiSeq4000 (Paired-end). Data processing, alignment (using a Burrows-Wheeler algorithm, BWA-mem) and variant calling were performed using Genome Analysis Tool Kit (GATK v4) best practices (https://software.broadinstitute.org/gatk/best-practices/) from the Broad Institute. Variant annotation was done using ANNOVAR (http://www.openbioinformatics.org/annovar/). Variants were picked up in exonic and splicing regions with a minor allele frequency of ≤0.005 in SNP database (ExAC_EAS, ExAC_ALL, 1000Genomes, gnomAD). The identified variants were confirmed and segregation analysis of the two variants from parents was applied by Sanger sequencing.

**Fig. 2:**
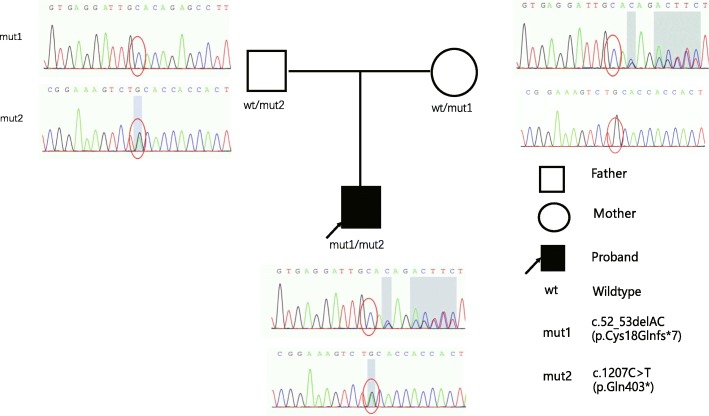
Whole-exome sequencing (WES) and Sanger sequencing revealed compound heterozygous truncating variants c.1207C > T (p.Gln403*), c.52_53delAC (p.Cys18Glnfs*7)) in *AP4B1*(NM_001253852.2). Electrochromatograms illustrated the variants in patient (arrow) and his parents. wt, wildtype

We detected two heterozygous truncating variants (c.1207C > T (p.Gln403*), c.52_53delAC (p.Cys18Glnfs*7)) in *AP4B1*(NM_001253852.2) from patient (Fig. 2). His parents were heterozygous carriers. The patient inherited c.1207C > T allele from his father and c.52_53delAC from his mother. Both variants were suggested to be pathogenic according to the American College of Medical Genetics and Genomics and the Association for Molecular Pathology (ACMG-AMP) variant interpretation guidelines [9]. These compound heterozygous truncating variants were considered to be disease-causing in the boy with the clinical symptoms of spastic tetraplegia, moderate psychomotor development delay and febrile seizures plus.

**Fig. 3:**
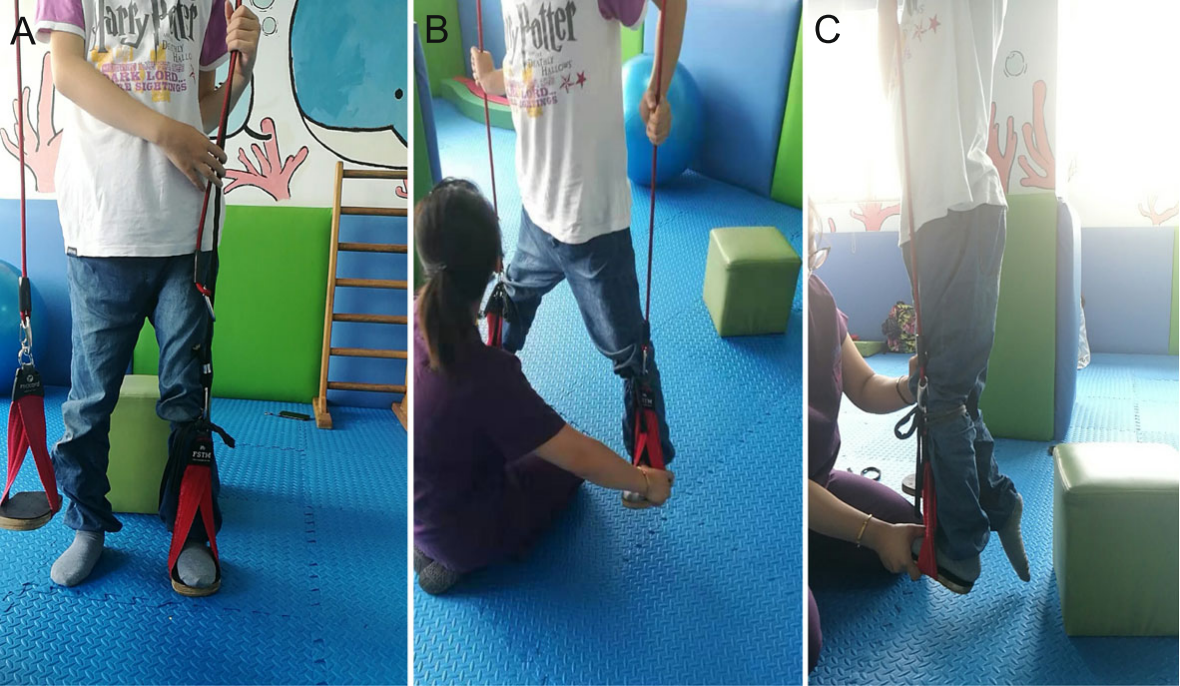
Photos of physical therapy of the patient. **a** Unilateral lower limb resistance standing. **b** Autonomous outreach training on slings. **c** Autonomous Achilles tendon stretching training

The boy started on rehabilitating program with physical therapy on regular basis. The detailed regimens were as following: kinesitheraphy (30 min/time, 4 times/week), sling exercise therapy (30 min/time, 2 times/week), continue passive motion (20 min/time, 4 times/week). Joint stretching, joint activity training and progressive resistance training were mainly used to enhance the separation of the lower limbs, core muscles and the lower limbs-pelvis-torso. They were also used to correct the abnormal gait (Fig. 3). In addition, psycho-social support was also an important part of the treatment. He showed significant improvement after 2 years of program judging by the angle changes of dorsiflexion, popliteal fossa, and adductors as well as the evaluation data on Gross Motor Function Measure (GMFM) (Table 1). An improvement on active and passive foot dorsiflexion angle, popliteal fossa angle and adductor angle reflected the progress of limb function in patient (Table 1). The increase in GMFM score suggested that the patient’s gross motor function has not regressed (in theory), but progressed continuously over time, proving the effectiveness of treatment.

**Table 1:** Examination of rehabilitation effects

	201705^b^	201709	201801	201906
Dorsiflexion Angle(active)				
left	−10°	0°	0°	25°
Right	-10°	0°	0°	15°
Dorsiflexion Angle(passive)				
left	−5°	15°	15°	45°
Right	−5°	15°	15°	25°
Popliteal fossa Angle				
left	90°	90°	97°	100°
Right	85°	90°	95°	100°
Adductors Angle	80°	90°	90°	105°
GMFM^a^	82.13	84.96	86.45	90.58

^a^*GMFM* Gross Motor Function Measure

^b^ Date for physical test

## Discussion and conclusions

We reported a patient with spastic tetraplegia, moderate psychomotor development delay and febrile seizures plus-. A paternal heterozygous nonsense variant c.1207C > T (p.Gln403*) and a maternal heterozygous frameshift variant c.52_53delAC (p.Cys18Glnfs*7) which resulted in the introduction of a premature termination codon in two different alleles were identified in *AP4B1* gene. Ebrahimi-Fakhari et al [5] reported the clinical and genetic characterization of nineteen probands with *AP4B1*-associated SPG47 including early developmental delay and intellectual disability(100%), delayed motor development(100%), neonatal or infantile hypotonia(100%), delayed speech development(94%), progression to spastic diplegia (89%), loss of independent walking (88%), short stature (57%), thin corpus callosum(73%), delayed myelination or white matter loss(67%), ventriculomegaly(40%). Only half of the patients had epilepsy (47%), especially febrile seizures (3/19). Symptoms of the patient we reported here were consistent with the clinical characterizations of patients reported previously. Accogli et al. [6] reported another SPG47-related child who was admitted to the hospital at the age of 14 months. The child had an afebrile generalized tonic-clonic epilepticus status which required resuscitation. Our patient, who had been diagnosed as febrile seizures plus, had afebrile seizures more than 2 times before developing typical febrile seizures. He also continued febrile seizures beyond the age of 6 years. This condition was a relatively rare feature reported before.

AP4 is a heterotetrameric adaptor protein which composed of two large subunits, beta-4 (AP4B1) and epsilon-4 (AP4E1), one medium protein, mu-4 (AP4M1), and one small protein, sigma-4 (AP4S1)(10). Besides of *AP4B1*, mutations on other three subunits can also cause autosomal recessive-HSPs. Homozygous or compound heterozygous mutations in *AP4M1* result in autosomal recessive spastic paraplegia-50 (SPG50, MIM:612936) is characterized by neonatal hypotonia that progresses to hypertonia and spasticity and severe mental retardation with poor or absent speech development [10, 11]. *AP4E1* and *AP4S1* are related to SPG51 (MIM:613744) and SPG52 (MIM:614067) respectively with the similar symptoms [4, 12, 13]. Hardies et al. [14] reported two sisters, born of unrelated Caucasian parents, who showed clinical features including developmental delay, febrile seizures, and spastic paraplegia caused by *AP4S1*. The older sister had five brief generalized febrile seizures between 5 months and 5 years of age whose manifestation was similar to our patient.

**Fig. 4:**
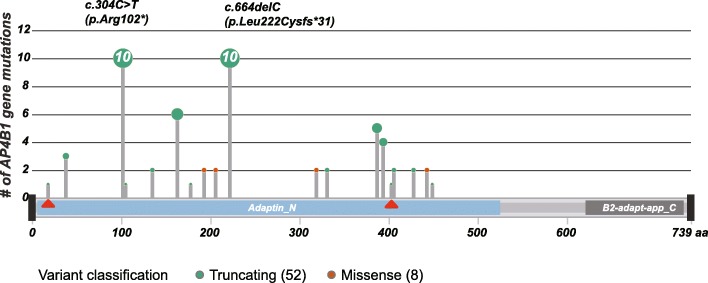
Lollipop graph shows mutations in *AP4B1* gene reported in literatures. Red triangles indicate the two variants identified in our patient

Totally, twenty-two mutants in *AP4B1* have been reported including the ones from our patient (Supp Table 1). Homozygous mutations c.304C > T (p.Arg102*) and c.664delC (p.Leu222Cysfs*31) are the most frequently detected variants from consanguineous families. The allele counts are 10 for each of two mutations from 30 patients (Fig. 4). The vast majority of pathogenic variants identified so far are truncating variants (allele counts ratio is 52/60) which can often be assumed to disrupt gene function by leading to a complete absence of the gene product by nonsense-mediated decay of an altered transcript or lack of transcription (e.g. nonsense, frameshift, canonical splice site).

The patient has little progress through medications and rehabilitations. His seizures are well controlled by adjusted medication of anti-epileptic drug, 12.5 mg/kg (Bid) of levetiracetam tablets. He remained seizure-free for more than 2 years. No apparent regression had been seen in patient’s motor development by physical therapy, including joint stretching, joint activity training and progressive resistance training. His speech and language development had been severely delayed for a long time. He could speak a few words (“baba”, “mama”) until 2-year-old. We thought it might have a relationship with seizures to some extent, therefore, we paid more attention to anti-epileptic treatment clinically. For language and speech impairment, the hospital’s teaching and parent intervention methods were used due to the need to protect the children’s other daily activities. Our department keeps on providing follow-up care at home regularly and adjusting the guidance program according to the situation of patient. He is supported and cared by relatives, friends as well as the whole society. In conclusion, in this report we identified two novel pathogenic variants from a Chinese patient with clinical features of hereditary spastic paraplegias, including spastic tetraplegia, moderate psychomotor development delay and febrile seizures plus. Our findings expanded the knowledge of genotypic and phenotypic heterogeneity and similarity of HSPs. Early discovery, diagnosis and proper treatment on the conditions generally increase chances of improvement on the quality of life for patients.

## Supplementary information

**Additional file 1: Supplemental Table1.** Mutants in AP4B1 gene collected in research papers.

## Data Availability

The datasets generated during the current study are available in NCBI SRA, under the accession number “SRR11117837”.

## Abbreviations

ACMG-AMP: American College of Medical Genetics and Genomics and the Association for Molecular Pathology
FS: Febrile seizures
GMFM: Gross Motor Function Measure
HSPs: Hereditary spastic paraplegias
SPG47: Autosomal recessive spastic paraplegia-47
